# The contrasting responses of abundant and rare microbial community structures and co-occurrence networks to secondary forest succession in the subalpine region

**DOI:** 10.3389/fmicb.2023.1177239

**Published:** 2023-05-12

**Authors:** Xiaoying Zhang, Wenqiang Zhao, Yongping Kou, Kai Fang, Yanjiao Liu, Heliang He, Qing Liu

**Affiliations:** ^1^CAS Key Laboratory of Mountain Ecological Restoration and Bioresource Utilization & Ecological Restoration and Biodiversity Conservation Key Laboratory of Sichuan Province, Chengdu Institute of Biology, Chinese Academy of Sciences, Chengdu, China; ^2^University of Chinese Academy of Sciences, Beijing, China; ^3^Faculty of Agriculture, Forestry and Food Engineering, Yibin University, Yibin, China

**Keywords:** subalpine secondary forest succession, abundant microbial community, rare microbial community, co-occurrence network, keystone taxa

## Abstract

Knowledge of variations in abundant and rare soil microbial communities and interactions during secondary forest succession is lacking. Soil samples were gathered from different secondary successional stages (grassland, shrubland, and secondary forest) to study the responses of abundant and rare bacterial and fungal communities, interactions and driving factors to secondary forest succession by Illumina sequencing of the 16S and ITS rRNA genes. The results showed that the *α*-diversities (Shannon index) of abundant bacteria and fungi revealed no significant changes during secondary forest succession, but increased significantly for rare bacteria. The abundant and rare bacterial and fungal *β*-diversities changed significantly during secondary forest succession. Network analysis showed no obvious changes in the topological properties (nodes, links, and average degree) of abundant microbial networks during secondary forest succession. In contrast, these properties of the rare microbial networks in the secondary forest were higher than those in the grassland and shrubland, indicating that rare microbial networks are more responsive to secondary forest succession than abundant microorganisms. Additionally, rare microbial networks revealed more microbial interactions and greater network complexity than abundant microbial networks due to their higher numbers of nodes and links. The keystone species differed between the abundant and rare microbial networks and consisted of 1 and 48 keystone taxa in the abundant and rare microbial networks, respectively. Soil TP was the most important influencing factor of abundant and rare bacterial communities. Successional stages and plant richness had the most important influences on abundant and rare fungal communities, respectively. C:P, SM and N:P were mainly related to abundant and rare microbial network topological properties. Our study indicates that abundant and rare microbial communities, interactions and driving factors respond differently to secondary forest succession.

## Introduction

Secondary succession refers to a sequential replacement of plant species over time or due to a natural or human forces disturbance (Horn, 1974). In recent years, large tracts of forest have undergone artificial or natural restoration stages due to the disturbance of human activities (i.e., logging and abandoned agricultural fields) and natural factors (i.e., insect outbreaks and wildfire) (Chai et al., 2019; Rodriguez-Ramos et al., 2021; Zhang X. et al., 2022). Previous studies have found that secondary forest succession is an effective way to improve soil conditions and restore degraded environments (Yan et al., 2020; Hu et al., 2022). Plant communities change during secondary forest succession, resulting in variations in soil microbial communities (Cline and Zak, 2015; Chai et al., 2019; Liu et al., 2020). Thus, elucidating the responses of soil microorganisms to secondary forest succession is essential for understanding the mechanism of forest development (Jiang et al., 2021). Numerous studies have found that the diversity and composition of soil microbial communities differ with secondary forest succession (Cline and Zak, 2015; Liu et al., 2020; Yan et al., 2020; Jiang et al., 2021; Zhang X. et al., 2022; Zhang et al., 2023). The variations in soil microbial community composition are connected to plant composition (Li et al., 2020), soil nutrient cycling (Li et al., 2020; Jiang et al., 2021; Zhang X. et al., 2022; Zhang et al., 2023), litter decomposition (Cline and Zak, 2015), plant disease resistance (Hu et al., 2020) and other ecosystem processes.

It is known that the distribution of microbial communities is usually skewed in natural ecosystems; there are relatively few abundant species with high relative abundance and numerous rare species with low relative abundance (Shade et al., 2014; Lynch and Neufeld, 2015; Jousset et al., 2017). The changes in abundant species can obscure low-abundance taxa dynamics; thus, abundant species are traditionally thought to participate in major ecological functions, while rare microorganisms are usually ignored (Lynch and Neufeld, 2015; Zhou and Wu, 2021). However, recent studies have indicated that rare microorganisms serve as vital contributors to microbial *α*diversity and *β*diversity (Lynch and Neufeld, 2015) and play a major role in driving the multifunctional stability of ecosystems (Jousset et al., 2017; Shu et al., 2021; Wu et al., 2021; Zhang Z. et al., 2022). For example, Wu et al. (2021) found that rare microbial communities indirectly regulate the impact of land degradation on ecosystem functions. Shu et al. (2021) reported that rare microorganisms are the main drivers of the soil nutrient cycle (i.e., C, N, and S cycling). Recently, numerous studies have focused on the responses of abundant and rare microbial communities to environmental change (Jiang et al., 2019; Du et al., 2020; Jiao and Lu, 2020; He Z. et al., 2022; Zhang Z. et al., 2022). The results of these studies showed that abundant and rare microbial communities exhibited distinct changes along environmental gradients and demonstrated different contributions to soil ecosystem multifunctionality. However, knowledge of how the abundant and rare microbial communities respond to secondary forest succession is still unclear.

Network analysis has been extensively adopted for investigating the interactions between microbial communities, which can also provide more information about the correlations between microbial interactions and ecosystem functions (de Vries et al., 2018; Wagg et al., 2019; Shi et al., 2020). In microbial networks, positive and negative correlations represent cooperative and competitive interactions between microbial communities, respectively (Faust and Raes, 2012; Xue et al., 2022). Population dynamics can be stabilized by a moderate mixture of cooperative and competitive interactions (Mougi and Kondoh, 2012). Furthermore, the increase in microbial interactions represents higher network complexity and microbial community stability (Mougi and Kondoh, 2012; Guo et al., 2019) and reveals more resistance to disturbance in the external environment (i.e., drought) (Wang et al., 2018; Yu et al., 2021). Recent studies have found that microbial network complexity varied in salt marsh succession (Dini-Andreote et al., 2014), abandoned cropland succession (Morrien et al., 2017), grassland succession (Guo et al., 2019) and forest succession (de Araujo et al., 2018; Hu et al., 2022). Moreover, sporadic research has also focused on abundant and rare microbial networks. For example, Jiang et al. (2019) found that abundant bacterial taxa exhibited closer relationships than rare bacterial taxa in bacterial networks in glacier forefield succession. The nodes of the abundant bacterial network mainly consisted of Proteobacteria and Acidobacteria, while Planctomycetia, Sphingobacteriia, and Phycisphaerae were dominant in the bacterial network. In addition, network analysis has also been used to identify potential keystone species by network-based scores (He et al., 2017; Wang et al., 2018; Yu et al., 2021). These keystone species have been reported to play unique and important roles in maintaining microbial community structures and ecosystem functions (Faust and Raes, 2012; Banerjee et al., 2018a; Zheng et al., 2021).

Both abundant and rare microbial communities were affected by biotic (i.e., plant richness) and abiotic factors (Jiang et al., 2019; He Z. et al., 2022; Pan et al., 2022). For instance, Jiang et al. (2019) found that plant richness, soil organic carbon (SOC) and pH mainly affected abundant bacterial communities, while soil total nitrogen (TN) and pH primarily affected rare bacterial communities during glacier forefield succession. He Z. et al. (2022) found that soil electrical conductivity (EC), available nitrogen (AN), nitrate (NO_3_^−^-N), available phosphorus (AP) and land-use type had extremely significant influences on abundant bacteria, while soil pH, EC, soil water content (SM), AP and land-use type had extremely significant influences on rare bacteria in reforestation succession soil. In addition, abiotic factors have also been reported to have important influences on microbial interactions. For example, soil bulk density, altitude, soil moisture (Ji et al., 2022), SOC (Dong et al., 2022), pH (Banerjee et al., 2018b; Dong et al., 2022; Ji et al., 2022), NH_4_^+^-N, C:N and organic phosphorus (Banerjee et al., 2018b) have been reported to exert major functions in regulating soil microbial interactions. Understanding the driving factors of soil microbial communities and interactions during succession is crucial. However, how the changes in environmental factors (i.e., soil properties and plant richness) affect abundant and rare microbial communities and interactions during secondary forest succession remains unclear.

The subalpine forest in the southwest is regarded as the major body of alpine forests on the eastern Qinghai-Tibet Plateau, which is situated in the upper reaches of the Yangtze River. This area has a vital effect on water resource conservation, regional economy and ecological shelter (Liu et al., 2001). This area gradually displayed different recovery stages of secondary forest (grassland–shrubland–secondary coniferous forest) after large-scale deforestation, which provided an ideal platform for studying the succession of subalpine forests (Qiang et al., 2021). Sporadic studies in this area have revealed changes in microbial community compositions (Cao et al., 2020; Qiang et al., 2021; Xu et al., 2021; Zhang X. et al., 2022; Zhang et al., 2023), functions (Zhang X. et al., 2022; Zhang et al., 2023), and interactions (Qiang et al., 2021; Zhang et al., 2023). Our previous studies in this area also revealed that soil microbial communities and functions change significantly during secondary forest succession (Zhang X. et al., 2022; Zhang et al., 2023). However, it is still unknown how the abundant and rare microbial communities and their interactions respond to secondary forest succession, which limits our understanding of the mechanism of secondary forest succession. As a result, the present study was aimed at exploring the variations in abundant and rare microbial community structures, interactions and driving factors during secondary forest succession. We hypothesized that 1) abundant and rare microbial communities respond differently to secondary forest succession; 2) abundant and rare microbial interactions are inconsistent in response to secondary forest succession; 3) the influencing factors of abundant and rare microbial communities and interactions are different during secondary forest succession.

## Materials and Methods

### Site description and sampling

The study site is located at the Miyaluo Nature Reserve of Sichuan Province (ranged from 31°42′ to 31°51’N, ranged from 102°41′ to 102°44′E, 2200 to 5,500 m a.s.l.). The region is characterized by a warm summer and a dry winter with a snowy climate. With the annual rainfall precipitation ranging from 600 mm/y to 1,100 mm/y, the mean annual temperature is shown to be 8.7°C. In addition, the soil type in this area is mountain brown soil. This area gradually formed a natural recovery sequence (from grassland to secondary forest) after being extensively deforested from 1918 to 1998 (Liu et al., 2001; Liu, 2002). In this study, the early, middle, and late successional stages of the natural recovery sequence were selected to investigate the responses of abundant and rare microbial communities and interactions to secondary forest succession. Specifically, the grassland stage represents the early stage of forest recovery (approximately 0–20 years) and is dominated by *Poa annua*; the shrubland stage represents the middle stage of forest recovery (approximately 30 years) and is dominated by *Quercus aquifolioides*, followed by *Picea asperata*, *Sorbus koehneana* and *Betula albosinensis*; the secondary forest represents the late stage of forest recovery (approximately 60 years) and is dominated by *Picea asperata*, followed by *Quercus aquifolioides* and *Betula platyphylla*. The three selected successional stages were similar in climate, slope and geology.

In August 2018, we set up approximately 100 m × 100 m sampling areas in grassland, shrubland and secondary forest. For sampling rhizosphere and bulk soil, this study established five plots of grassland (1 m × 1 m), shrubland (5 m × 5 m) and secondary forest (10 m × 10 m). In order to avoid edge effects and spatial autocorrelation, the plots of each successional stage were more than 20 m apart (Chen et al., 2019). Detailed sampling information and sampling methods have been published in our recent papers (Zhang X. et al., 2022; Zhang et al., 2023). Briefly, the rhizosphere and bulk soils were collected by root shaking methods and soil drilling (Zhang X. et al., 2022), respectively. The 5 rhizosphere and bulk soil samples collected from each plot were combined as one composite soil. Finally, 30 soil samples were collected at the three successional stages. These soil samples were classified into the following two parts after passing through a 2 mm sieve: one part was maintained at 4°C for soil physical and chemical property analysis; besides, the other part was kept at −40°C for soil DNA extraction.

### Soil physicochemical properties

Soil EC and pH were determined with a soil-to-water ratio of 1:2.5 by a DDS307 conductivity meter and Mettler-Toledo FE28 digital pH meter, respectively. We calculated SM by drying soil samples for 72 h at 65°C. SOC and TN were measured with a 2400II CHN elemental analyser (PerkinElmer, Boston, MA, United States). The measurement of ammonium (NH_4_^+^-N) and NO_3_^−^-N was performed using the indophenol blue method as well as the phenol disulfonic acid method following extraction with 2 M KCl, respectively. Total phosphorus (TP) was calculated by molybdenum antimony blue colorimetry after digestion with HClO_4_-H_2_SO_4_. Using the ratio of SOC and TN, the soil C:N was calculated; C:P was calculated by the ratio of SOC and TP, and N:P was calculated by the ratio of TN and TP. All of the soil properties were previously published in Zhang X. et al. (2022).

### DNA extraction, high-throughput sequencing, and data processing

Using MoBio PowerSoil DNA Isolation Kits (MoBio Laboratories, CA, United States), total DNA was extracted from each soil sample. We evaluated the integrity of the extracted DNA by 1.0% (*w*/*v*) agarose gel electrophoresis. In addition, with a NanoDrop NC 2000 spectrophotometer (Thermo Scientific), the quality of the DNA extracts was calculated. To amplify the ITS2 region of fungi (Ihrmark et al., 2012), the primer set gITS7F (5′-GTGARTCATCGARTCTTTG-3′) and ITS4R (5′-TCCTCCGCTTATTGATATGC-3′) was used, and the primer set 515\u00B0F 5′-TATCGCCTCCCTCGCGCCATCAG-3′ and 909 R 5′-CTATGCGCCTTGCCAGCCCGCT-3′ was applied to amplify the V4-V5 region of the bacterial 16S rRNA gene (Tamaki et al., 2011). More details regarding the PCR system and conditions for fungal and bacterial PCR amplification were given by Zhang X. et al. (2022) and Zhang et al. (2023), respectively. Then, the amplicon samples were subjected to paired-end sequencing on the Illumina HiSeq (PE 250 × 250) platform at Biomarker Technologies Corporation (Beijing, China). The high quality sequences were clustered at the 100% similarity level. To avoid biases of the different sequencing depths, random resampling was carried out at the number of sequences 10,065 and 8,000 for bacteria and fungi, respectively. On the basis of the Silva v138.1 database and UNITE^1^ or fungal ITS database (Nilsson et al., 2019), the representative sequences of bacterial and fungal species were classified. The raw sequencing data were submitted to the National Center for Biotechnology Information (NCBI) SRA database (accession number: PRJNA835217 and PRJNA733908) and have been reported in our previous studies (Zhang X. et al., 2022; Zhang et al., 2023).

### Network construction with a random matrix theory-based approach

Molecular Ecological Networks Pipeline (MENAP)^2^ was used to illustrate the ecological interactions within microbial communities (Zhou et al., 2010; Deng et al., 2012). Initially, following the operational standards of the MENAP, the standardized ASV table was uploaded. ASVs with a relative abundance >0.1% were regarded as abundant ASVs, while ASVs with a relative abundance <0.01% were determined to be rare ASVs (Jiao and Lu, 2020; Wan et al., 2021). More than 50% of the total samples of abundant and rare microbial taxa were used for microbial network analysis at each successional stage. The missing values were filled with 0.01 in blanks with paired valid values. This correlation matrix was transformed into a similarity matrix by taking the absolute values. Then, thresholds from 0.30 to 1.00 (with an interval of 0.01) were adopted until an optimal similarity threshold was identified where significant non-random patterns were detected in the microbial networks (Wang et al., 2018). To compare abundant and rare microbial networks among different successional stages, an identical cut-off of 0.88 was used to construct the microbial networks. For the purpose of depicting the characteristic parameters of the microbial network, nodes, links, path length, the clustering coefficient and modularity were used in this study. A detailed description of these parameters can be found in (Deng et al., 2012). The visualization of networks was conducted in Cytoscape 3.8.0 (Shannon et al., 2003). The nodes with *Zi* ≤ 2.5 and *Pi* ≤ 0.62 can be regarded as peripheral nodes; connectors were determined by *Zi* ≤ 2.5 and *Pi* > 0.62; module hubs were characterized by *Zi* > 2.5 and *Pi* ≤ 0.62; besides, and network hubs were identified as *Zi* > 2.5 and *Pi* > 0.62.

### Statistical analysis

One-way analysis of variance (ANOVA) and Tukey’s HSD test were conducted to analyse the differences (*p* < 0.05) in plant richness and soil properties among different successional stages by SPSS software (Version 19.0, SPSS Inc., Chicago, IL, United States). The Wilcoxon test was used to identify the differences in abundant and rare microbial *α*-diversities among different successional stages. Principal coordinate analysis (PCoA) based on Bray–Curtis distance was conducted to compare the abundant and rare microbial communities among different successional stages. One-way analysis of similarity (ANOSIM) was used to identify the differences in abundant and rare microbial communities between different successional stages. The partial Mantel test with 999 permutations was used to calculate the relationships between abundant and rare microbial communities and environmental factors based on Pearson correlation (*p* < 0.05) with the “vegan” package. The Pearson correlation analysis was used to analyse the correlations between environmental factors and microbial network topological properties. Partial least squares path modeling (PLS-PM) was conducted to identify the complex relationships between forest succession, plant richness, soil properties and microbial communities with SmartPLS 4 software.

## Results

### Abundant and rare microbial diversities and community compositions among different successional stages

**Figure 1:**
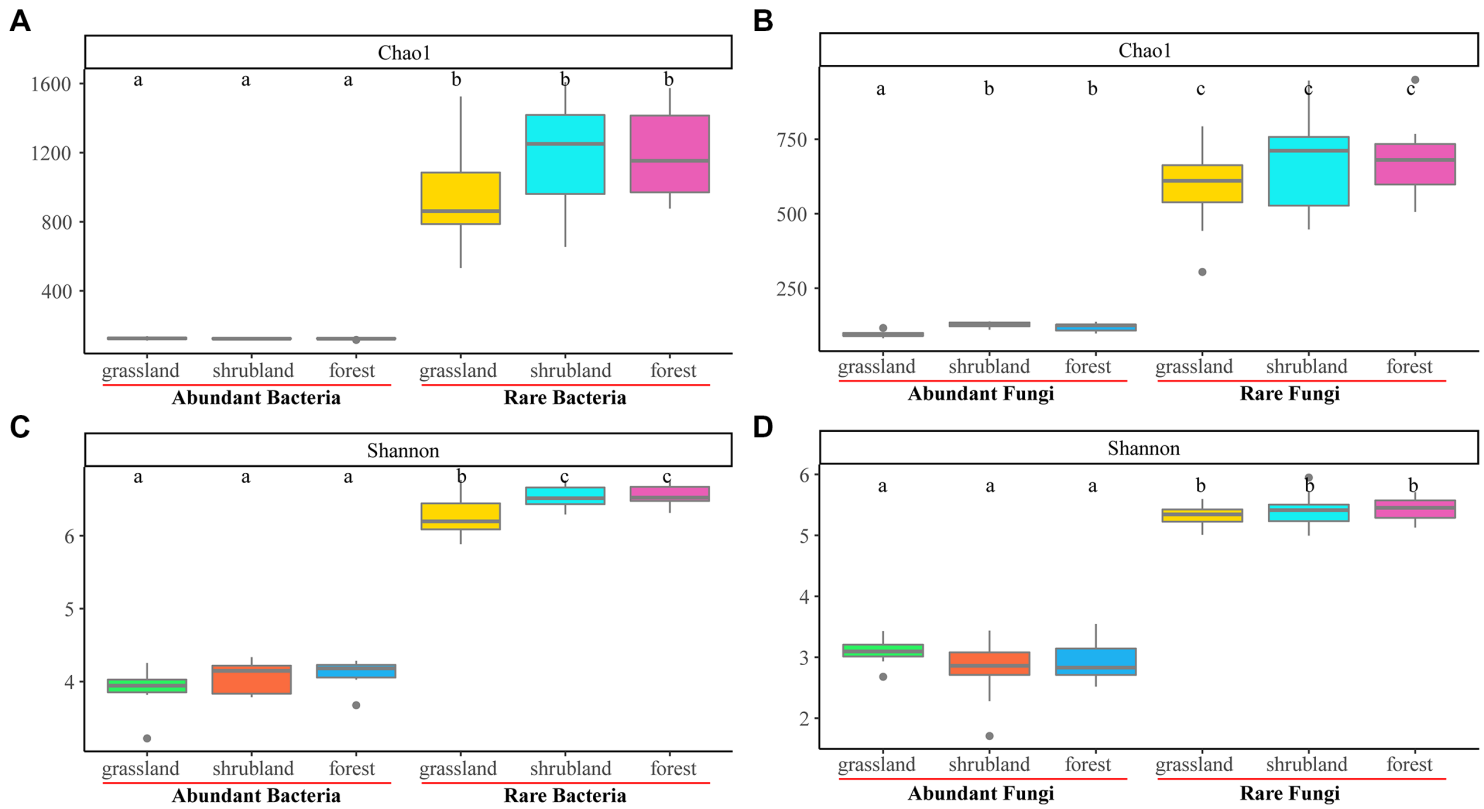
Boxplot of the Chao1 and Shannon index of abundant and rare bacterial communities **(A, C)**, and abundant and rare fungal communities **(B, D)** during secondary forest succession.

**Figure 2:**
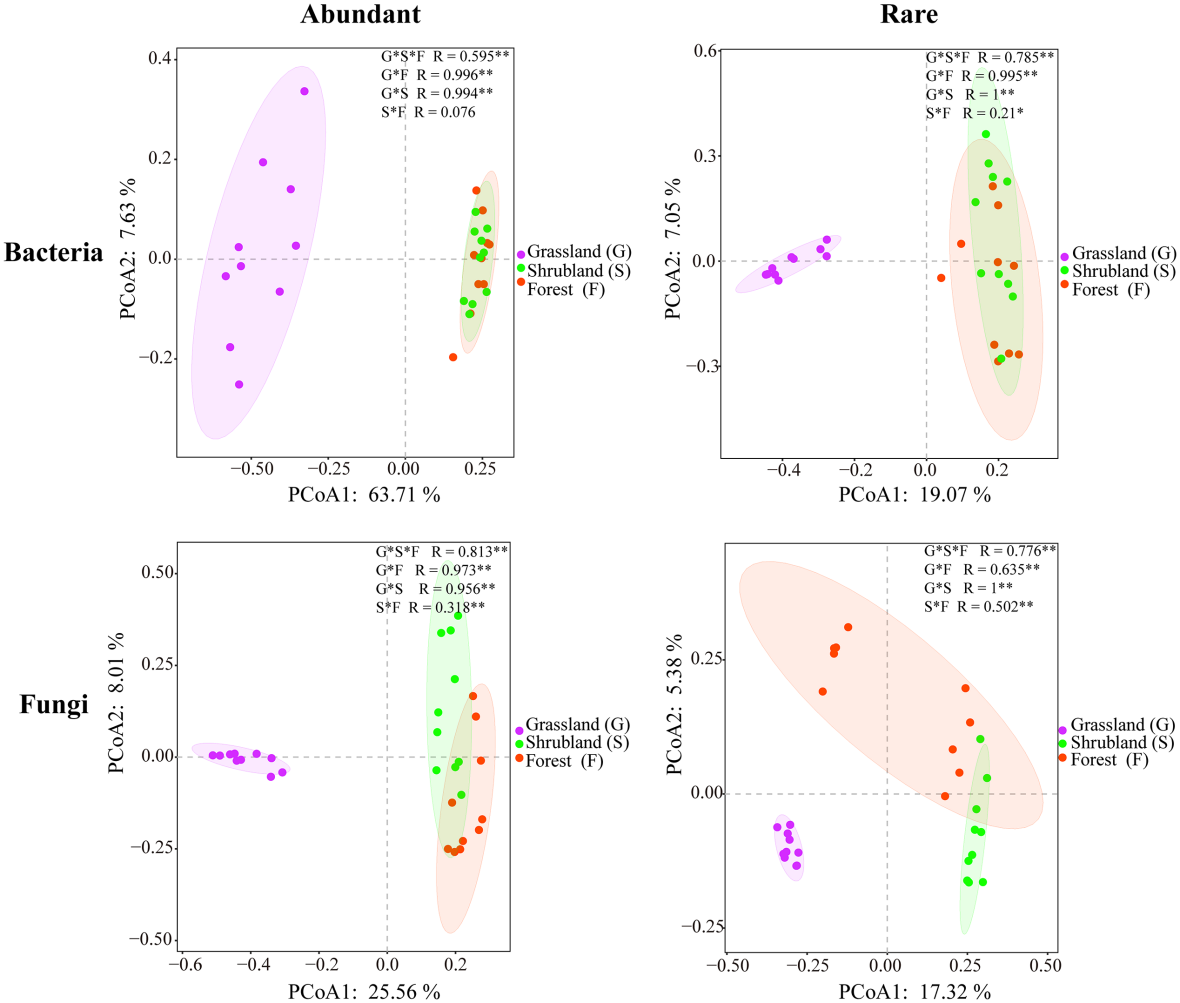
Principal coordinate analysis according to the Bray–Curtis distances showing the abundant and rare microbial communities.

Abundant bacterial Chao 1 and Shannon index and the rare bacterial Chao 1 index showed no significant changes during secondary forest succession (*p* > 0.05), while the rare bacterial Shannon index of shrubland and secondary forest was significantly higher than that in the grassland (*p* < 0.05) (Figures 1A,C). The abundant fungal Shannon index and rare fungal Chao 1 and Shannon index revealed no significant difference (*p* > 0.05), while the abundant fungal Chao 1 index of shrubland and secondary forest was significantly higher than that in the grassland (*p* < 0.05) (Figures 1B,D). Moreover, the Chao 1 and Shannon index of abundant bacteria and fungi were significantly lower than those of rare bacteria and fungi (*p* < 0.05) (Figures 1A-D). The *β*-diversities of abundant bacteria and fungi (*R* = 0.595, *p* = 0.001; *R* = 0.813, *p* = 0.001) and rare bacteria and fungi (*R* = 0.785, *p* = 0.001; *R* = 0.776, *p* = 0.001) changed significantly with secondary forest succession (Figure 2).

**Figure 3:**
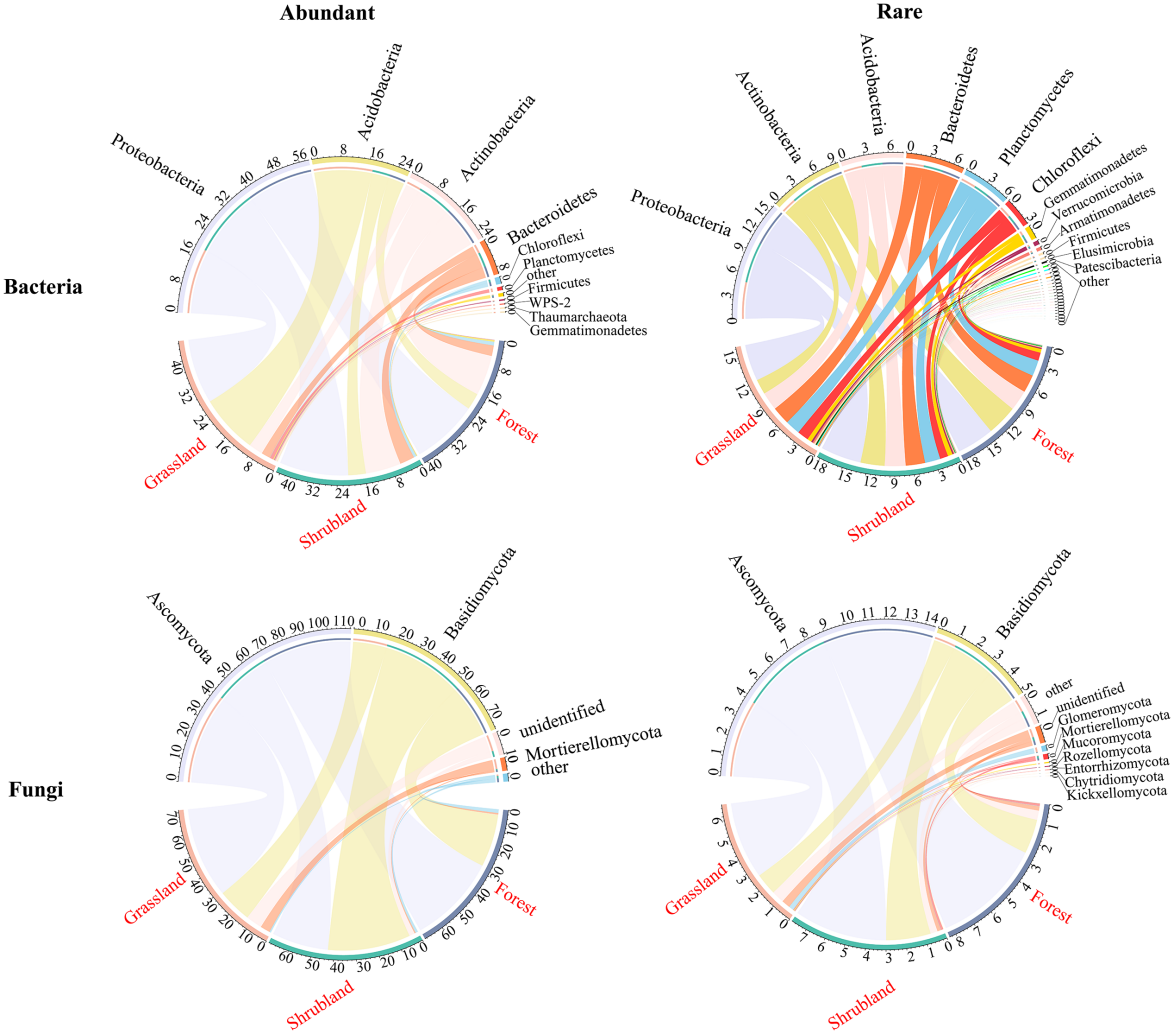
Chord diagram of abundant and rare bacterial and fungal community compositions at the phylum level among the three successional stages.

A total of 7,263 bacterial ASVs were detected in all samples, and abundant and rare bacterial taxa included 166 ASVs and 5,671 ASVs, respectively. The numbers of abundant and rare bacterial phyla were 10 and 29 at the three successional stages, respectively (Figure 3). The abundant and rare bacterial communities were dominated by Proteobacteria (18.97–20.18%, 4.86–5.41%), Acidobacteria (4.82–17.20%, 2.43–2.78%) and Actinobacteria (4.16–11.80%, 2.00–4.07%) (Figure 3). A total of 3,971 fungal ASVs were identified in all samples, and abundant and rare fungal taxa included 181 ASVs and 3,043 ASVs among the three successional stages, respectively. The numbers of abundant and rare fungal phyla were 4 and 10 at the three successional stages, respectively (Figure 3). Ascomycota (26.34–42.31%, 3.99–5.71%) and Basidiomycota (15.93–38.58%, 1.12–2.30%) were the dominant phyla of abundant and rare fungal communities (Figure 3).

### Co-occurrence networks of abundant and rare microbial communities among different successional stages

**Figure 4:**
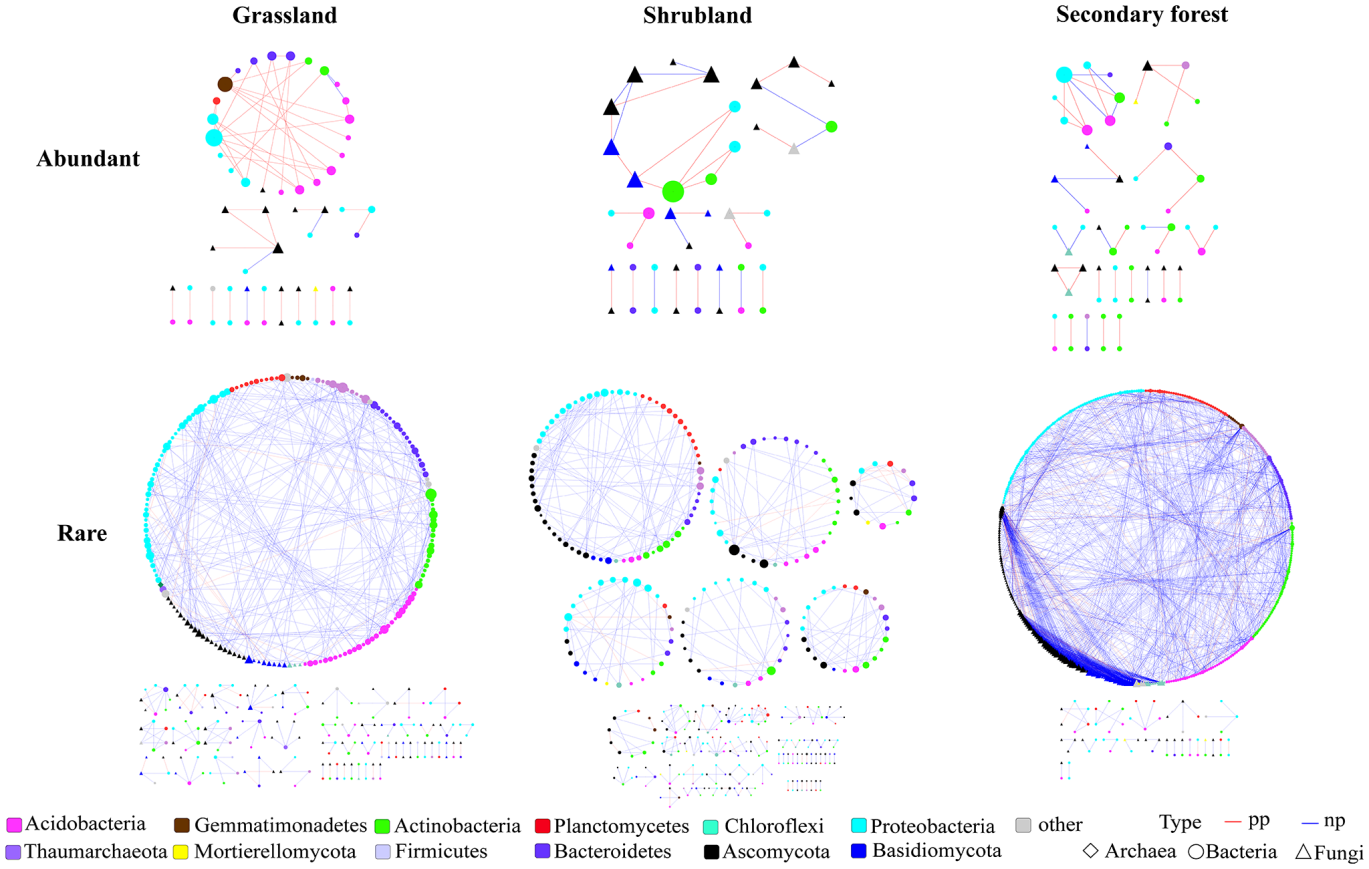
Co-occurrence network analysis of the abundant and rare microbial communities during forest successional stages.

According to the network analysis results, the modularity of abundant and rare microbial networks was >0.4 (Table 1). The nodes, links, and average degree of the abundant microbial network showed no obvious changes with succession (Table 1). In contrast, the nodes, links, transitivity, density, and average degree of the rare microbial network in the secondary forest were higher than those in the grassland and shrubland, while the modularity of the rare microbial network revealed a contrary trend (Table 1). Furthermore, the numbers of nodes and links, average degree, average path distance, and harmonic geodesic distance of the abundant microbial networks were lower than those of the rare microbial networks at the three successional stages (Table 1). Additionally, our findings demonstrated that the transitivity of the abundant microbial network was higher than that of the rare microbial network at the three successional stages (Table 1). Furthermore, the nodes of abundant and rare microbial networks were dominated by bacteria (except for in the abundant microbial network in the shrubland) rather than fungi (Figure 4).

**Table 1:** Topologicacl properties of the abundant and rare microbial networks as well as their random networks during different successional stages.

		Abundant			Rare		
	Network features	Grassland	Shrubland	Secondary forest	Grassland	Shrubland	Secondary forest
Empirical networks	Threshold	0.88	0.88	0.88	0.88	0.88	0.88
	Total nodes	56	41	58	352	395	458
	Total links	48	32	42	386	538	1,651
	Negative correlations	5 (10.42%)	10 (31.25%)	12 (28.57%)	352 (91.19%)	395 (73.42%)	1,553 (94.06%)
	Positive correlations	43 (89.58%)	22 (68.75%)	30 (71.43%)	34 (8.81%)	143 (26.58%)	98 (5.94%)
	R square of power-law	0.969	0.882	0.973	0.881	0.862	0.709
	Average degree (avgK)	1.714	1.561	1.448	2.193	2.538	7.21
	Average clustering coefficient (avgCC)	0.039	0.138	0.118	0.02	0.036	0.082
	Average path distance (GD)	3.332	2.26	1.605	7.421	8.587	6.055
	Harmonic geodesic distance (HD)	2.332	1.596	1.331	5.439	6.805	4.813
	Centralization of degree (CD)	0.1	0.064	0.065	0.022	0.022	0.083
	Centralization of betweenness (CB)	0.087	0.023	0.009	0.064	0.14	0.093
	Density (D)	0.031	0.039	0.025	0.006	0.006	0.016
	Transitivity (Trans)	0.038	0.387	0.333	0.02	0.037	0.129
	Connectedness (Con)	0.182	0.094	0.046	0.251	0.603	0.748
	Module	17	13	20	60	65	33
	Modularity	0.752	0.791	0.903	0.904	0.946	0.542
Random networks	Average clustering coefficient (avgCC)	0.020 ± 0.012	0.036 ± 0.010	0.026 ± 0.009	0.004 ± 0.003	0.004 ± 0.003	0.091 ± 0.008
	Average path distance (GD)	4.180 ± 0.644	3.193 ± 0.715	2.744 ± 0.585	6.622 ± 0.228	6.253 ± 0.118	3.229 ± 0.030
	Harmonic geodesic distance (HD)	3.028 ± 0.376	2.195 ± 0.379	1.905 ± 0.277	5.607 ± 0.154	5.451 ± 0.085	2.952 ± 0.020
	Transitivity (Trans)	0.022 ± 0.026	0.024 ± 0.042	0.013 ± 0.033	0.008 ± 0.005	0.006 ± 0.003	0.142 ± 0.005
	Modularity(fast_greedy)	0.750 ± 0.020	0.778 ± 0.031	0.861 ± 0.026	0.780 ± 0.009	0.714 ± 0.007	0.299 ± 0.004

### Keystone species of abundant and rare microbial networks among different successional stages

**Figure 5:**
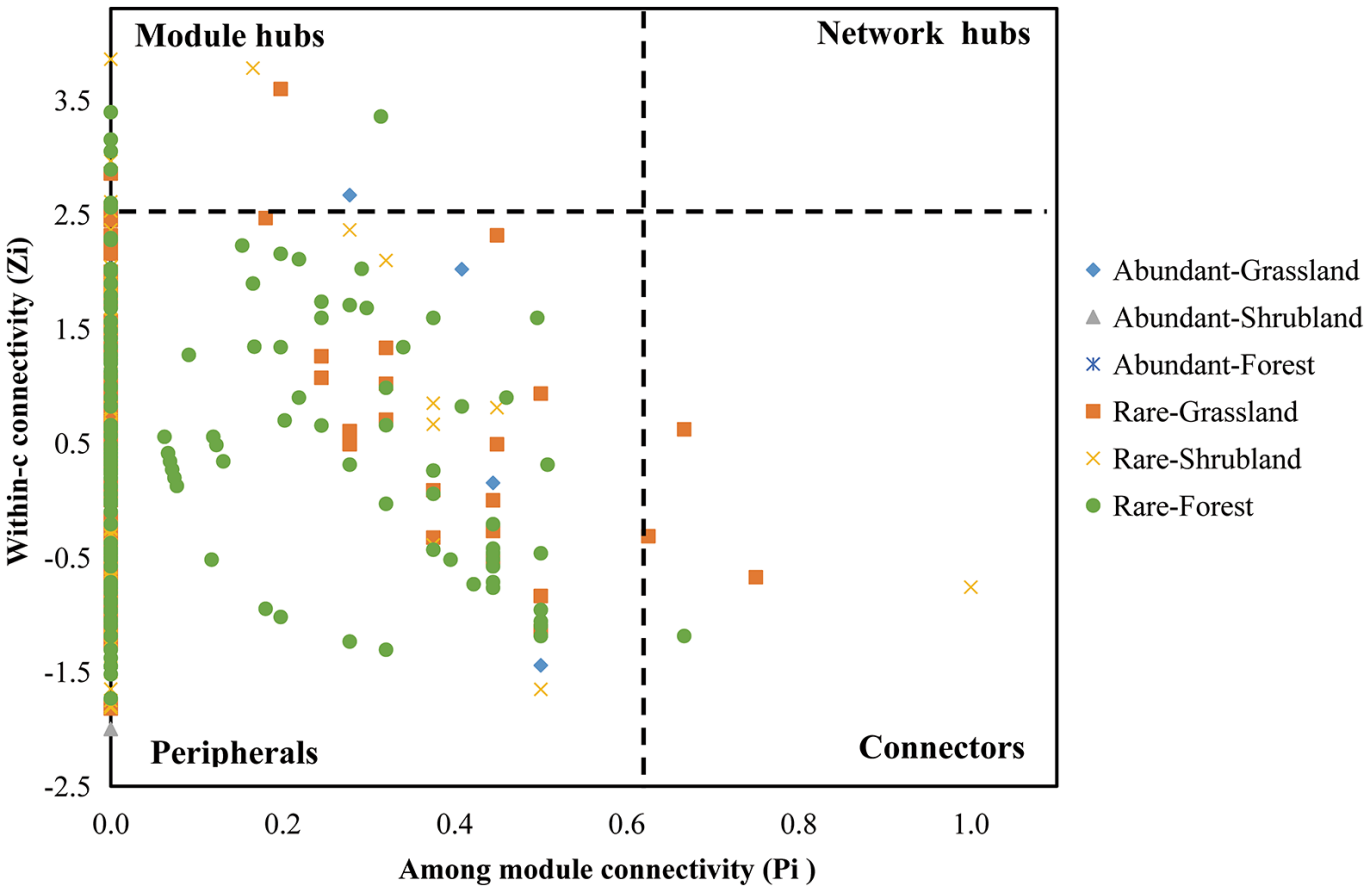
Putative keystone species of abundant and rare microbial networks within forest succession. Based on the scatter plot of among-module connectivity (*Pi*) and within-module connectivity (*Zi*), the topological role of each ASV was identified. In addition, the threshold values of *Zi* and *Pi* for categorizing ASVs were found to be 2.5 and 0.62, respectively.

Previous studies have noted that module hubs, connectors and network hubs could play the role of putative keystone species (He et al., 2017; Wang et al., 2018; Yu et al., 2021). In the current study, we found that the numbers of keystone species in the abundant microbial networks were lower than those in the rare microbial networks. Specifically, the abundant microbial networks consisted of 1 keystone taxa (Gemmatimonadetes, *Gemmatimonas*). The rare microbial networks consisted of 49 keystone taxa, including 5 in Acidobacteria (Acidobacteriales, Acidobacteria bacterium SCN 69–37, Subgroup 6 and *Bryobacter*), 4 in Actinobacteria (Ilumatobacteraceae, uncultured actinobacterium, IMCC26256 and *Jatrophihabitans*), 2 in Bacteroidetes (Microscillaceae and uncultured *Flexibacter* sp.), 1 in Chloroflexi (Elev-1,554), 1 in Gemmatimonadetes (Gemmatimonadaceae), 2 in Planctomycetes (Gemmataceae and *Zavarzinella*), 10 in Proteobacteria (Acetobacteraceae, Diplorickettsiaceae, R7C24, Betaproteobacteriales, SC-I-84, Rhizobiales, Myxococcaceae, *Caulobacter* and *Bradyrhizobium*), 1 in Verrucomicrobia (*Opitutus*), 17 in Ascomycota (Helotiales, Helotiaceae, GS34, Capnodiales, *Venturia*, *Knufia*, *Leohumicola*, *Oidiodendron*, *Phialocephala* and *Podospora*), 2 in Basidiomycota (*Hymenogaster* and *Mycena*) and 4 king taxa (Figure 5).

### Driving factors of abundant and rare microbial community compositions and network topological properties

**Figure 6:**
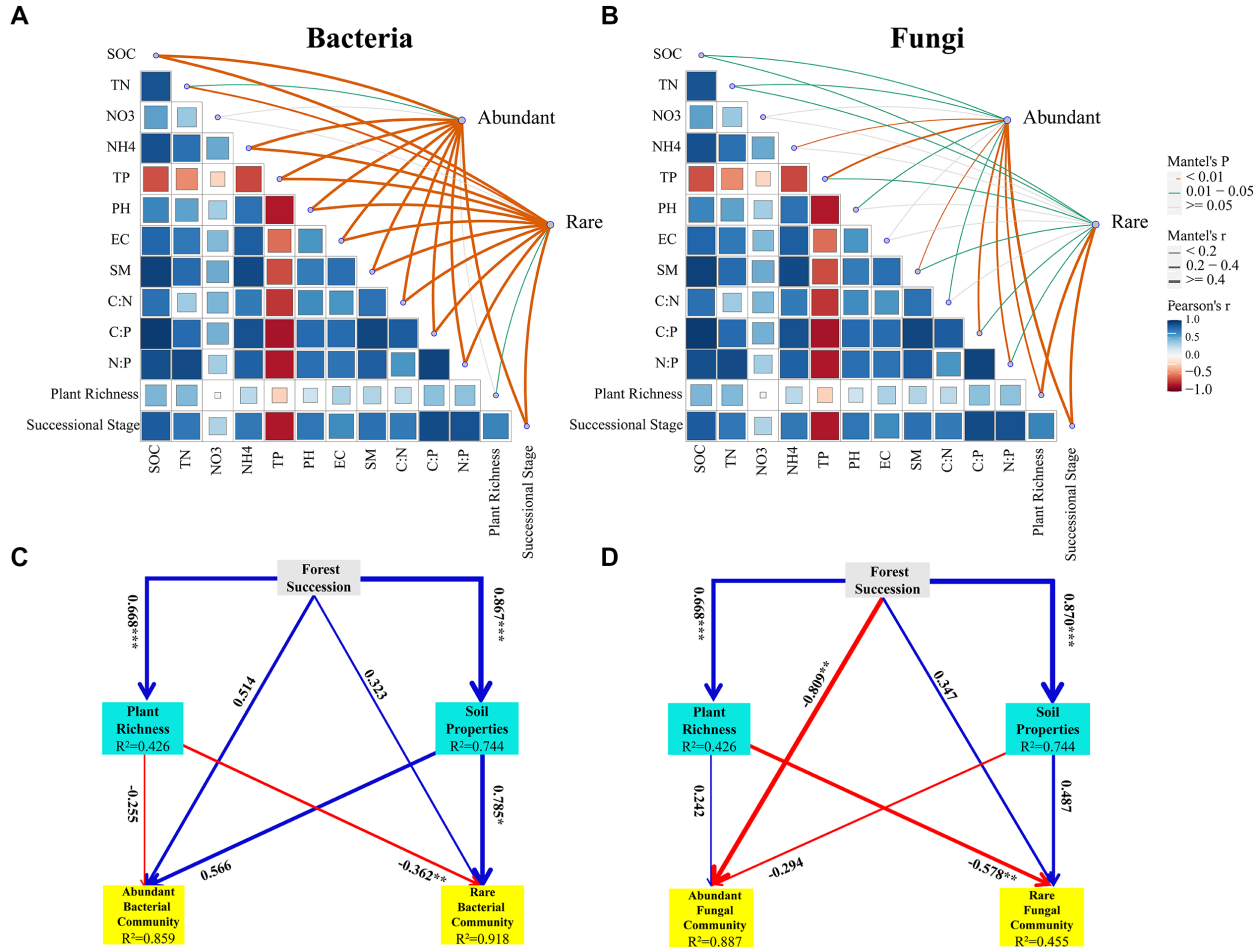
Pairwise comparisons between the abundant and rare bacterial **(A)** and fungal communities **(B)** in response to environmental factors during secondary forest succession. Pearson’s correlation coefficients of abundant and rare bacterial and fungal taxa and environmental factors in accordance with Mantel tests. Edge width is consistent with Mantel’s r value, and the colour of the edge suggests the statistical significance on the basis of 999 permutations. Partial least-squares path model (PLS-PM) of selected environmental factors for abundant and rare bacterial **(C)** and fungal **(D)** communities during different successional stages. The value of path coefficients are shown arrow width, blue and red colors indicate positive and negative effects, respectively. SOC, soil organic carbon; TN, total nitrogen; NO3, nitrate nitrogen; NH4, ammonium nitrogen; TP, total phosphorus; EC, electrical conductivity; SM, soil moisture content; C:N, soil organic carbon to total nitrogen; C:P, soil organic carbon to total phosphorus; N:P, total nitrogen to total phosphorus.

A partial Mantel test was used to analyse the correlations between environmental factors (successional stages, plant richness and soil properties) and abundant and rare bacterial and fungal communities. As shown in the results, all the soil factors (except for NO_3_^−^-N) had significant influences on the abundant and rare bacterial communities (*p* < 0.05) (Figure 6A). Plant richness had a significant influence on the rare bacterial community (*p* < 0.05) but no significant influence on the abundant bacterial community (*p* > 0.05). Among them, soil TP was the most important factor affecting abundant (*r* = 0.89, *p* = 0.001) and rare (*r* = 0.79, *p* = 0.001) bacterial communities (Figure 6A). For the fungal community, soil properties (SOC, TN, TP, SM, C:P and N:P), successional stage and plant richness were significantly correlated with abundant and rare fungal communities (*p* < 0.05) (Figure 6B). NH_4_^+^-N, pH and C:N presented strong associations with abundant fungal communities rather than rare fungal communities. Successional stage (*r* = 0.42, *p* = 0.001) and plant richness (*r* = 0.44, *p* = 0.001) were the most vital influencing factors of the abundant and rare fungal communities, respectively (Figure 6B).

The PLS-PM results revealed that the path model explained 85.9 and 91.8% of the variances in the abundant and rare bacterial *β*-diversities, respectively (Figure 6C). Plant richness had a negative effect on the abundant (path coefficient = −0.255) and rare (path coefficient = −0.362) bacterial *β*-diversities (Figure 6C). Soil properties had a positive effect on the abundant (path coefficient = 0.566) and rare (path coefficient = 0.785) bacterial *β*-diversities (Figure 6C). For soil fungi, the path model explained 88.7 and 45.5% of the variances in the abundant and rare fungal *β*-diversities, respectively (Figure 6D). Plant richness had a positive effect on the abundant fungal *β*-diversity (path coefficient = 0.242), but had a negative effect on the rare fungal *β*-diversity (path coefficient = −0.578) (Figure 6D). Soil properties had a negative effect on the abundant (path coefficient = −0.294) fungal *β*-diversity, while it had a positive effect on the rare fungal *β*-diversity (path coefficient = 0.487) (Figure 6D).

**Figure 7:**
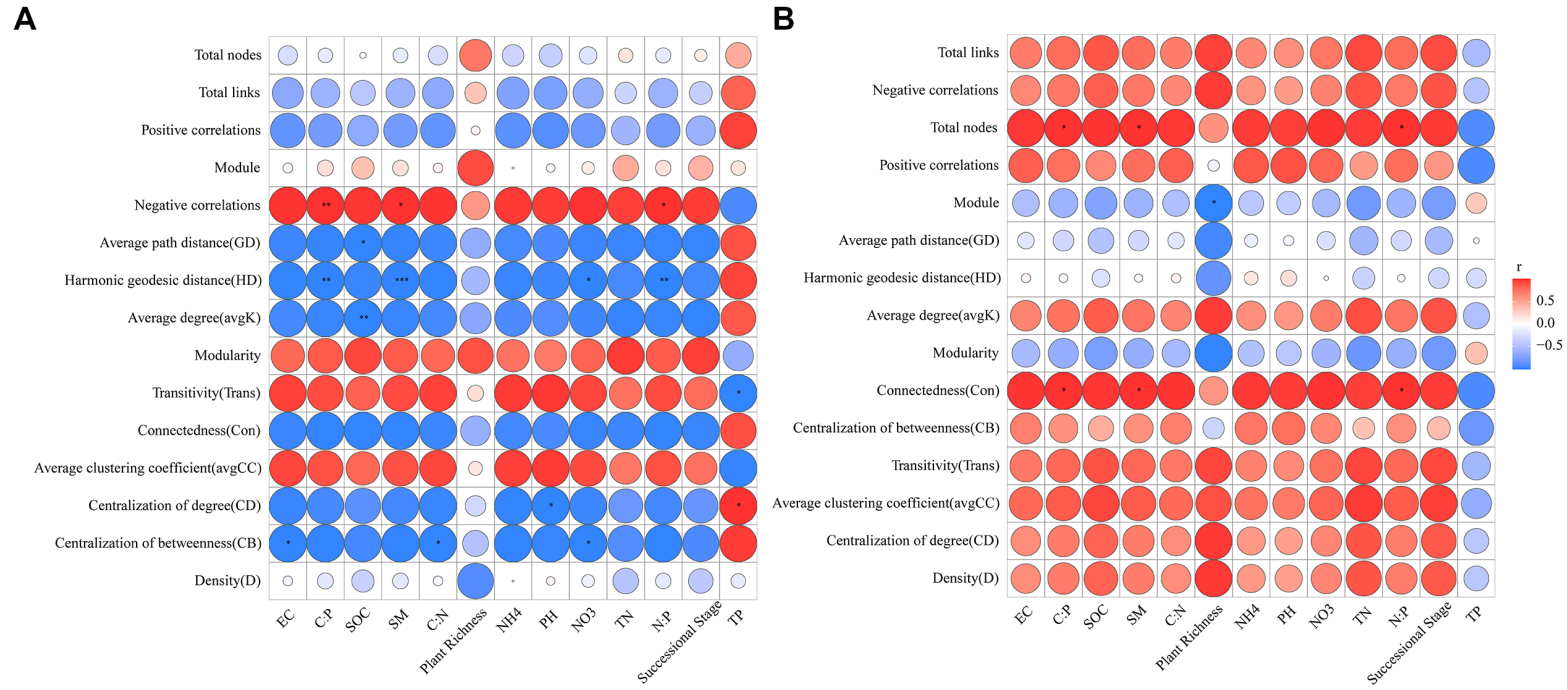
Pearson correlations between abundant **(A)** and rare **(B)** microbial network topological properties and environmental factors. SOC, soil organic carbon; TN, total nitrogen; NO3, nitrate nitrogen; NH4, ammonium nitrogen; TP, total phosphorus; EC, electrical conductivity; SM, soil moisture content; C:N, soil organic carbon to total nitrogen; C:P, soil organic carbon to total phosphorus; N:P, total nitrogen to total phosphorus.

The results of Pearson correlation analysis showed that C:P, SM, and N:P were significantly positively correlated with negative correlations of the abundant microbial networks but significantly negatively correlated with the harmonic geodesic distance (*p* < 0.05) (Figure 7A). SOC was negatively related to the average degree and average path distance of the abundant microbial networks (*p* < 0.05) (Figure 7A). C:P, SM and N:P were positively correlated with connectedness and total nodes of the rare microbial networks (*p* < 0.05) (Figure 7B). Plant richness was negatively related to the module of the rare microbial networks (*p* < 0.05) (Figure 7B).

## Discussion

### Abundant and rare microbial communities exhibit distinct changes during secondary forest succession

Previous studies have found that biotic factors (i.e., plant traits and plant richness) (Chai et al., 2019; Qiang et al., 2021) and soil factors (Qiang et al., 2021; Zhang X. et al., 2022; Zhang et al., 2023) drive changes in microbial diversity and community composition during secondary forest succession. In this study, we found significant changes in the abundant and rare bacterial and fungal *β*-diversities during secondary forest succession (*p* = 0.001). This was in line with previous studies in different ecosystems (Jiang et al., 2019; Du et al., 2020; Shu et al., 2021). This could be related to the significant changes in plant richness and soil factors (i.e., SOC, TN, NH_4_^+^-N, NO_3_^−^-N, C:N, C:P and N:P) (Table S1, S2). Moreover, the *α*-diversities of the abundant bacterial and fungal communities were significantly lower than those of the rare communities, which was consistent with previous studies in reforestation succession (He Z. et al., 2022), salt marsh ecosystems (Du et al., 2020) and glacier forefield succession (Jiang et al., 2019). This result also supports the previous idea that rare taxa were the main contributors to microbial diversity (Lynch and Neufeld, 2015). In this study, the numbers oSf rare bacterial and fungal phyla were obviously higher than those of the abundant bacterial and fungal phyla. Proteobacteria was the main dominant bacterial phyla of abundant and rare bacteria. Proteobacteria species have high resource utilization and adaptability to external conditions (Goldfarb et al., 2011), thus showing a higher competitive advantage in the process of secondary forest succession. Ascomycota and Basidiomycota were dominant abundant and rare fungal phyla and were also the main soil fungi in forest soil (Jiang et al., 2018, 2021; Hu et al., 2022); they can participate in the decomposition of plant litter (Štursová et al., 2020; Dong et al., 2021; Zheng et al., 2021) and play significant roles in soil nutrient cycling during forest succession.

### Rare microbial co-occurrence networks are more responsive to forest succession than abundant microbial networks

Network analysis has been extensively adopted for elucidating microbial interactions as well as their responses to environmental changes (Deng et al., 2012; Dini-Andreote et al., 2014; Hu et al., 2022). In the current work, we first analysed the interactions of abundant and rare microbial communities within secondary forest succession in the subalpine area. Our results showed that the modularity of abundant and rare microbial networks during different successional stages was >0.4, indicating that microbial networks have modular structures during secondary forest succession (Xue et al., 2017; Hu et al., 2022). Some recent studies have found that abundant and rare microbial networks respond differently to environmental changes (Jiang et al., 2019; He Z. et al., 2022; Zhang X. et al., 2022). This is also supported by our study, we found that the network topological properties (i.e., nodes, links, and average degree) of the abundant microbial networks showed no obvious changes with secondary forest succession, while these properties of the rare microbial network in the secondary forest were higher than those in the grassland and shrubland, indicating that the rare microbial networks are more responsive to secondary forest succession than the abundant microbial networks. In addition, our results found that positive correlations were dominant in abundant microbial networks at different successional stages (Table 1), indicating stronger mutual cooperation interactions between abundant microorganisms, which is conducive to wider ecological niches and better environmental adaptation (Jiang et al., 2019; Du et al., 2020; Jiao and Lu, 2020; He G. et al., 2022). However, the rare microbial community was mainly negatively correlated, indicating that the competition among rare taxa was strong during the succession process, which resulted in a narrow niche of rare microbial communities (He Z. et al., 2022). In addition, we found that the numbers of nodes and links of the abundant microbial network were lower than those of the rare microbial networks at the three successional stages (Table 1). This suggests that rare microbial communities have more interactions and more complex microbial networks than abundant microbial communities. This result is similar to the findings obtained from reforestation succession soil (He Z. et al., 2022). Previous studies have confirmed that microbial network complexity is positively related to the versatility of ecosystems (Wagg et al., 2019). Therefore, the abundant and rare microbial community might have different influences on ecosystem function during secondary succession. Notably, in line with previous studies in a woodland-grassland ecotone (Banerjee et al., 2018b) and forest soil (Nakayama et al., 2019), we found that the nodes of the microbial networks were dominated by bacteria rather than fungi, which suggested that bacteria might play more important roles in maintaining the microbial network structure than fungi during secondary forest succession.

### Keystone species were different between the abundant and rare microbial networks during secondary forest succession

Keystone species are usually highly connected taxonomic groups and are important for microbial structure and function (Banerjee et al., 2018a; Zheng et al., 2021). The removal of keystone species leads to the decomposition of the microbial network (Zheng et al., 2021). Keystone communities have a vital effect on ecosystem functioning (Banerjee et al., 2018a; Zheng et al., 2021; Jin et al., 2022). For example, Zheng et al. (2021) and Jin et al. (2022) confirmed that keystone species certified by network analysis were involved in litter degradation through *in vitro* culturing experiments. In this study, the abundant keystone taxon *Gemmatimonas*, which plays a major role in nitrous oxide reduction due to product-specific nitrous oxide reductases (Park et al., 2017; Oshiki et al., 2022), was reported to be the keystone taxon in *Cunninghamia Lanceolata*-*Phoebe chekiangensis* mixed plantations (Ding et al., 2023). Moreover, *Gemmatimonas* could be the indicator bacteria in soil nutrient content changes (Liu et al., 2022) and is able to mineralize organophosphate and dissolve inorganic nitrogen, phosphorus and potassium (Xu et al., 2023). In addition, most of the rare keystone taxa have effects on plant growth and the nutrient cycle. In general, Gemmatimonadaceae, *Bryobacter* (Wang et al., 2022) and *Zavarzinella* (Ivanova et al., 2018) contribute to organic matter decomposition and nutrient cycling. Acetobacteraceae (Reis and dos Santos Teixeira, 2015) and *Bradyrhizobium* (Shahrajabian et al., 2021) are nitrogen fixation bacteria that can improve the organic nitrogen content to promote plant production. *Caulobacter* was also reported to enhance plant biomass (Berrios, 2022). *Oidiodendron* is an ericoid mycobiont that can form mycorrhizae with *Rhododendron* sp. and prompts plant growth by improving plant nitrogen absorption (Wei et al., 2016; Lin et al., 2020). *Mycena* is a mycorrhizal fungus and can enhance the growth of seedlings and roots (Zhang et al., 2012). *Phialocephala* is a widespread plant endophyte that can improve plant phosphorus nutrition (Mikheev et al., 2022). In contrast, *Venturia* has been widely reported as a plant pathogenic fungus, causing plant diseases such as apple scab (Bowen et al., 2011), olive leaf spot (Buonaurio et al., 2023), and pecan scab (Standish et al., 2021), which can adversely affect the health and growth of plants. Notably, some rare keystone taxa (i.e., *Jatrophihabitans*, *Knufia*, *Leohumicola*, *Hymenogaster* and *Podospora*) have rarely been studied. Thus, the ecological functions of these microbes are still unknown and need to be focused on in the future. Overall, our study explores the keystone species of abundant and rare microbial communities, providing a theoretical basis for an in-depth understanding of microbial community composition and interactions. Notably, network analysis does not always represent actual microbial interactions, so the keystone species identified by network analysis are statistical. The ecological function and importance of keystone taxa still require further experimental verification (Freilich et al., 2018; Banerjee et al., 2018a; Zheng et al., 2021).

### Driving factors of the abundant and rare microbial community compositions and co-occurrence networks

Investigating the driving factors of microbial community compositions and networks during secondary forest succession is crucial for understanding forest succession mechanisms. In this study, we considered the joint effects of successional stages, plant richness and soil properties on the abundant and rare microbial communities and networks during secondary forest succession. The results showed that secondary forest succession had both direct and indirect influences on the abundant and rare microbial community compositions (Figures 6C,D). Secondary forest succession was positively related to plant richness (*p* < 0.001) (Figures 6C,D). Plant richness increased with secondary forest succession and was conducive to improving soil carbon availability (Lange et al., 2015) and diversifying plant-derived available resources (Reynolds et al., 2003) for soil microorganisms, thereby regulating abundant and rare soil microbial community compositions during secondary forest succession. In addition, secondary forest succession had significantly positive influences on soil properties (i.e., SOC, TN, NH_4_^+^-N, NO_3_^−^-N, SM, EC, pH, C:N, N:P, and N:P) (*p* < 0.001) (Figures 6C,D), and the changed soil properties also drove the changes in abundant and rare microbial communities (Jiang et al., 2019; He Z. et al., 2022). The PLS-PM results showed that the interpretation rate of the joint effects of forest succession, plant richness and soil properties for the abundant fungal community was obviously higher than that of the rare fungal community, and most of the variations in the rare fungal community could not be explained by the selected environmental variables. A possible explanation for this result is that rare fungi were driven not only by environmental factors but also by stochastic population fluctuation processes and and fitness trade-offs (Jousset et al., 2017). Soil TP was the most important driving factor of abundant and rare bacterial communities during succession (*r* = 0.89, *p* = 0.001; *r* = 0.79, *p* = 0.001) (Figure 6A). This could be attributed to extensive *P* limitation in this study area (Yin et al., 2021). Previous studies in forest soils (Wan and He, 2020; Yan et al., 2021) also found that TP had an important impact on soil bacterial communities. In contrast, successional stages and plant richness were the most important influencing factors for the abundant and rare fungal communities, respectively (Figure 6B). The different influencing factors of bacteria and fungi during succession might be related to the following reasons: Soil fungi (i.e., mycorrhizal fungi) could form symbioses with specific plant species (Bai et al., 2020) and participate in lignin and cellulose decomposition of litter (i.e., white-rot fungi) (Rodríguez-Couto, 2017). The changes in plant richness during secondary forest succession result in changes in litter properties, which further lead to diversification of fungal litter decomposers. Thus, plant richness had a more important influence on the soil fungal community than on the soil bacterial community. Different from the driving factors of abundant and rare microbial communities during succession, soil C:P, SM, and N:P revealed significant correlations with the abundant and rare microbial network topological properties. These results suggest that soil C:P, SM, and N:P might play major roles in regulating the abundant and rare microbial networks. Taken together, our results confirm that the driving factors of abundant and rare microbial communities and interactions are different during secondary forest succession.

## Conclusion

In conclusion, our results showed that both the *β*-diversities of abundant and rare bacterial and fungal communities changed significantly during secondary forest succession. The *α*-diversities of rare bacterial and fungal communities were significantly higher than those of the abundant communities, indicating that rare taxa might be the main contributors to microbial *α*-diversities. In addition, rare microbial networks showed more microbial interactions and greater network complexity, and were more responsive to secondary forest succession than abundant microbial communities, indicating that abundant and rare microbial co-occurrence networks responded differently to secondary forest succession. The keystone species varied in abundant and rare microorganisms at the three successional stages, and the numbers of keystone taxa of rare microorganisms were higher than those of abundant microorganisms. These keystone species might participate in plant growth and nutrient cycle during secondary forest succession. Moreover, plant richness and soil properties jointly drove changes in abundant and rare microbial communities and co-occurrence networks during secondary forest succession. However, the main influencing factors of abundant and rare microbial communities and co-occurrence networks were different. This finding provides theoretical knowledge for deeply understanding the mechanism driving microbial dynamics in the process of secondary forest succession.

## Data availability statement

The original contributions presented in the study are included in the article/Supplementary material, further inquiries can be directed to the corresponding authors.

## Author contributions

QL, YK, and WZ designed this study. QL, YK, WZ, YL, and HH collected the soil samples. HH carried out the soil property measurement. XZ performed the data analysis and wrote the manuscript. QL, YK, and KF revised the manuscript. All authors contributed to the article and approved the submitted version.

## Funding

This work was financially supported by the National Natural Science Foundation of China (grant numbers 41930645 and 32171550); the Second Tibetan Plateau Scientific Expedition and Research Program (2019QZKK0303); the Youth Innovation Promotion Association of the Chinese Academy of Sciences (grant numbers 2021371); the Open Fund of Ecological Restoration and Biodiversity Conservation Key Laboratory of Sichuan Province, Chengdu Institute of Biology, Chinese Academy of Sciences (KXYSWS2202).

## Conflict of interest

The authors declare that the research was conducted in the absence of any commercial or financial relationships that could be construed as a potential conflict of interest.

## Publisher’s note

All claims expressed in this article are solely those of the authors and do not necessarily represent those of their affiliated organizations, or those of the publisher, the editors and the reviewers. Any product that may be evaluated in this article, or claim that may be made by its manufacturer, is not guaranteed or endorsed by the publisher.

## Footnotes

^1^ https://unite.ut.ee/

^2^ http://ieg2.ou.edu/MENA
