# Supplementary material for: The contrasting responses of abundant and rare microbial community structures and co-occurrence networks to secondary forest succession in the subalpine region
Source: Front Microbiol. 2023 May 12;14:1177239. doi: 10.3389/fmicb.2023.1177239 (PMC10213230; doi:10.3389/fmicb.2023.1177239)
Supplement: Supplementary file 1 [file Data_Sheet_1.docx]

**Supplementary Material**

# The contrasting responses of abundant and rare microbial community structures and co-occurrence networks to secondary forest succession in the subalpine region

**Author names and affiliations:** Xiaoying Zhang^a, b^, Wenqiang Zhao^a^, Yongping Kou^a, *^, Kai Fang^a^, Yanjiao Liu^a^, Heliang He^a, b, c^, Qing Liu^a, *^

*^a^ CAS Key Laboratory of Mountain Ecological Restoration and Bioresource Utilization & Ecological Restoration and Biodiversity Conservation Key Laboratory of Sichuan Province, Chengdu Institute of Biology, Chinese Academy of Sciences, Chengdu 610041, China*

*^b^ University of Chinese Academy of Sciences, Beijing 100049, China*

*^c^ Faculty of Agriculture, Forestry and Food Engineering, Yibin University, Yibin, 644000*

***Corresponding author:** Qing Liu

Telephone: +86-28-82890995

Fax: +86-28-82890288

E-mail: [liuqing@cib.ac.cn](mailto:liuqing@cib.ac.cn)

ORCID: 0000-0002-7046-0307

***Corresponding author:** Yongping Kou

Telephone: +86-28-82890619

1. mail: [kouyp@cib.ac.cn](mailto:kouyp@cib.ac.cn)

## **Fig. S1** Rarefaction curves of bacterial richness (a) and shannon (b), and fungal richness (c) and shannon (d) at the three successional stages.


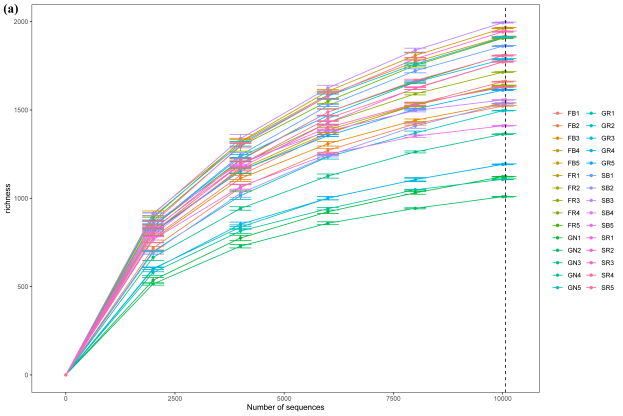

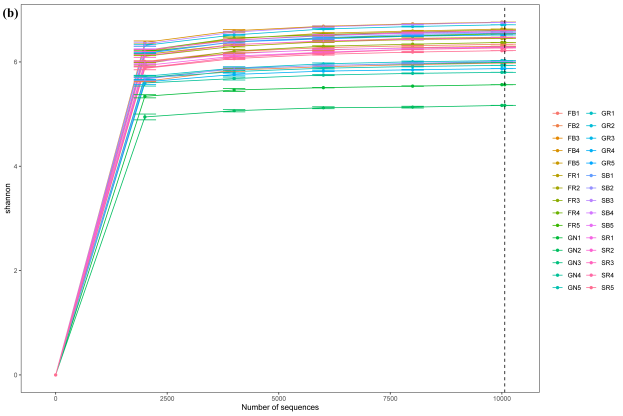


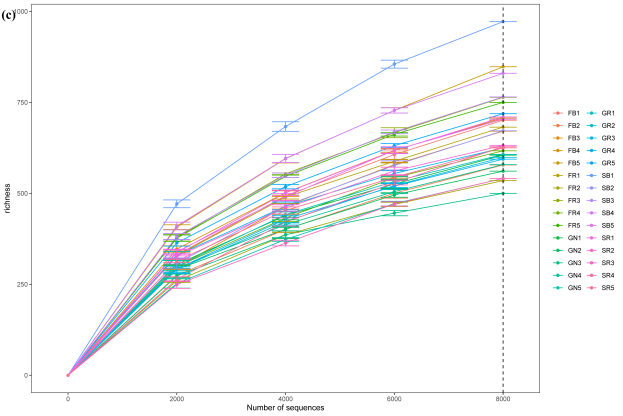

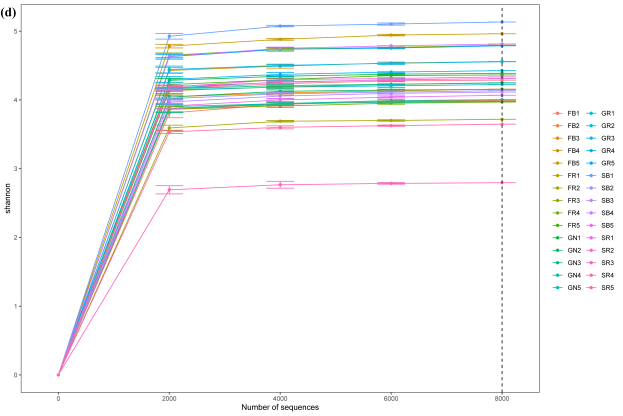


**Table S1** Plant richness at different successional stages.

| **Properties** | **Grassland** | **Shrubland** | **Forest** |
| --- | --- | --- | --- |
| **Plant Richness** | 10.40±0.75a | 8.40±1.66a | 18.20±0.58b |

Values are shown as means ± standard errors (n = 5). Different letters attached to the values indicate significant differences (*P* < 0.05).

**Table S2** Soil properties in the rhizosphere and bulk soils during the different successional stages. The soil properties were previously reported in Zhang et al. (2022a).

| **Soil Properties** | **GR** | **SR** | **FR** | **GB** | **SB** | **FB** |
| --- | --- | --- | --- | --- | --- | --- |
| **SOC (g/kg)** | 47.11±3.05b | 85.71±13.92ab | 130.04±15.19a | 37.37±2.53b | 97.27±8.97a | 127.18±15.44a |
| **TN (g/kg)** | 3.99±0.19bc | 5.06±0.77abc | 7.68±1.20a | 3.11±0.12c | 5.28±0.50abc | 6.42±0.42ab |
| **NO_3_^-^-N (mg/kg)** | 4.50±1.91a | 4.69±1.12a | 5.63±1.33a | 3.14±0.22a | 8.06±2.50a | 8.44±2.86a |
| **NH_4_^+^-N (mg/kg)** | 5.09±1.45b | 9.55±1.48ab | 11.54±1.53a | 2.69±0.29b | 11.83±0.89a | 11.15±0.98a |
| **TP (g/kg)** | 2.05±0.08a | 0.84±0.06bc | 0.96±0.04b | 1.91±0.04a | 0.87±0.04bc | 0.91±0.04b |
| **pH** | 5.74±0.02bc | 6.09±0.13ab | 6.21±0.05a | 5.60±0.05c | 6.34±0.07a | 6.29±0.09a |
| **EC (ms/cm)** | 103.32±9.19bcd | 121.82±17.47abc | 163.88±11.31a | 44.44±3.31d | 146.72±15.50ab | 127.90±17.81ab |
| **SM (%)** | 28.97±0.91bc | 55.21±6.54ab | 70.62±13.23a | 18.67±0.97c | 61.26±6.61ab | 70.76±10.15a |
| **C:N** | 11.88±0.81b | 16.82±0.93a | 17.77±1.90a | 12.05±0.79b | 18.46±0.69a | 19.66±1.63a |
| **C:P** | 23.03±1.42c | 99.60±9.62ab | 134.51±12.78a | 19.54±1.32c | 111.26±5.87a | 139.20±16.13a |
| **N:P** | 1.94±0.02c | 5.93±0.52ab | 7.90±1.10a | 1.62±0.05c | 6.05±0.37ab | 7.02±0.33a |

SOC, soil organic carbon; TN, total nitrogen; NO_3_^-^-N, nitrate nitrogen; NH_4_^+^-N, ammonium nitrogen; TP, total phosphorus; EC, electrical conductivity; SM, soil moisture content; C:N, soil organic carbon to total nitrogen; C:P, soil organic carbon to total phosphorus; N:P, total nitrogen to total phosphorus. GR and GB represent the rhizosphere and bulk soils of the grassland, respectively; SR and SB represent the rhizosphere and bulk soils of the shrubland, respectively; FR and FB represent the rhizosphere and bulk soils of the secondary forest, respectively. Values are shown as means ± standard errors (n = 5). Different letters attached to the values indicate significant differences (*P* < 0.05).
